# Strengthening of Vaccine-Preventable Disease (VPD) Surveillance to Enhance National Health Capacity and Security: Perspective from India

**DOI:** 10.3390/vaccines12080941

**Published:** 2024-08-22

**Authors:** Arun Kumar, Ratnesh Murugan, Satishchandra Donkatti, Deepa Sharma, Nirmal Kaundal, Tigran Avagyan, Pawan Kumar, Sunil Bahl, Sudhir Khanal, Vinod Bura

**Affiliations:** 1 World Health Organization, National Public Health Support Network, Country Office, New Delhi 110029, India; muruganr@who.int (R.M.); donkattis@who.int (S.D.); sharmade@who.int (D.S.); kaundaln@who.int (N.K.); avagyant@who.int (T.A.); 2 Ministry of Health and Family Welfare, Government of India, New Delhi 110011, India; drpawan.kumar@gov.in; 3 Public Health Expert, Formerly with World Health Organization, South-East Asia Region, New Delhi 110001, India; bahlsk@gmail.com; 4 World Health Organization, South-East Asia Region, New Delhi 110001, India; khanals@who.int (S.K.); burav@who.int (V.B.)

**Keywords:** surveillance, vaccine-preventable disease, acute flaccid paralysis, fever–rash, laboratory

## Abstract

The Government of India, in collaboration with the World Health Organization (WHO), established the National Polio Surveillance Project (NPSP) in 1997 and initiated acute flaccid paralysis (AFP) surveillance to achieve the goal of polio eradication. The WHO South-East Asia Region, comprising of 11 countries, including India, was certified as polio-free in March 2014. India was also validated to have eliminated maternal and neonatal tetanus in May 2015. Over the years, the surveillance of other vaccine-preventable diseases (VPDs) was integrated with AFP surveillance in the country. Outbreak-based measles–rubella (MR) surveillance was initiated in 2005 using AFP surveillance as a platform, case-based fever–rash (FR) surveillance started in 2021 as one of the strategies to achieve measles and rubella elimination in the country. The surveillance of diphtheria, pertussis, and neonatal tetanus was integrated with AFP surveillance in a phased manner during 2015–2022. The surveillance system for VPDs in India, supported by a laboratory network of 10 polio laboratories, 28 measles–rubella laboratories, and 20 diphtheria–pertussis laboratories, has enhanced the national health capacity and security. The setting up and expansion of the surveillance system in the country involved the important component of capacity building of personnel on various components of surveillance, including case identification, case investigation, sample collection and shipment, data analysis and public health response. These capacities have been used effectively during other emergencies, such as the recent COVID-19 pandemic, as well as during outbreaks of other diseases and natural calamities.

## 1. Introduction

Vaccine-preventable disease (VPD) surveillance is defined as the routine ongoing collection, analysis, and dissemination of health data of vaccine-preventable diseases which includes the following functions: the detection and notification of health events, collection and consolidation of pertinent data, investigation and confirmation of VPDs, routine analysis and creation of reports, and feedback of information to persons providing data [1]. VPD surveillance is critical for monitoring the overall immunization program performance. The evidence generated through surveillance helps to identify outbreaks and take appropriate public health measures, guide the introduction of new vaccines, detect changes in disease epidemiology, and monitor the status of disease elimination or eradication.

The universal immunization program (UIP) of India is one of the largest routine immunization programs in the world. The annual birth cohort of the country is nearly 26 million. The diseases for which vaccines are provided under UIP are tuberculosis, polio, diphtheria, pertussis, tetanus, hepatitis B, *H. influenzae*, rotavirus, pneumococcus, measles, rubella, and Japanese Encephalitis (Table 1) [2].

In India, the Immunization Division of the Ministry of Health and Family Welfare is responsible for the management of the immunization program and, with the technical support of the World Health Organization’s National Public Health Support Network (WHO-NPSN), is also responsible for the quality and breadth of VPD surveillance in the country.

The Government of India, in collaboration with the World Health Organization, established the National Polio Surveillance Project (NPSP) in 1997 to support the case-based, laboratory-supported acute flaccid paralysis (AFP) surveillance system as one of the strategies to achieve polio eradication [4]. A polio laboratory network was also established in the same year. The environmental surveillance for poliovirus detection was initiated in 2001 at three sites in Mumbai as a supplement to the AFP surveillance system. The number of sites for environmental surveillance was expanded over the years, and currently, there exist 63 sites in 32 cities of 14 states.

**Figure 1:**
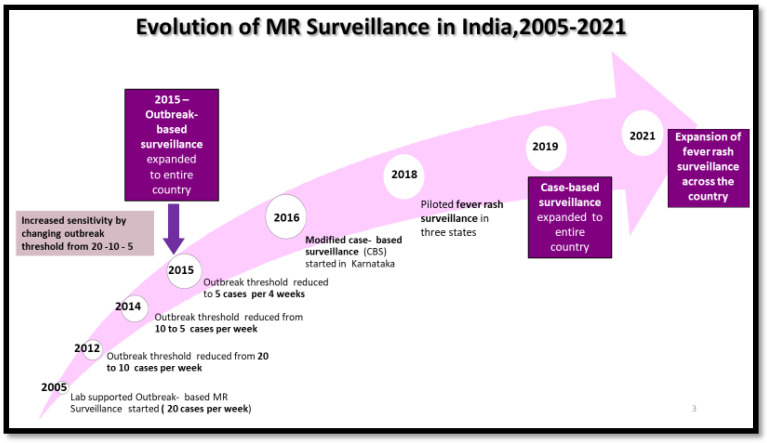
Evolution of MR surveillance system in India.

The expansion of the AFP surveillance system to cover a broader range of infectious diseases was a critical part of the founding vision back in 1996. Building upon the existing AFP surveillance system of the polio eradication initiative, India initiated an outbreak-based measles–rubella (MR) surveillance system in a few states in 2005 and expanded this to all states by 2015. The country transitioned from an outbreak-based to case-based MR surveillance between 2016 and 2019. Beginning in 2018, fever–rash (FR) surveillance was piloted in three states of the country (Karnataka, Madhya Pradesh, and Odisha), and based on the findings from this pilot study, FR surveillance expanded to the entire country in 2021 wherein the suspected case definition was broadened to include all cases of fevers with maculopapular rashes (Figure 1). These efforts helped in increasing the sensitivity of measles–rubella surveillance in the country [5].

In 2015, the government mandated the WHO-NPSP to support laboratory-supported case-based diphtheria–pertussis–neonatal tetanus (DPT) surveillance in the country. The objective of this surveillance was to generate evidence to guide the immunization program of the country. This surveillance was added to the existing AFP-MR surveillance system in 2015 and was gradually expanded to all states of the country by August 2022 [5,6].

It is important to note that in 2004, the Government of India initiated an ‘Integrated Disease Surveillance Project—IDSP’ and included several VPDs, such as cholera, diphtheria, hepatitis A, influenza, JE, measles, meningococcal meningitis, mumps, pertussis, pneumococcal disease, polio, rubella, typhoid, and varicella, under this project. The main purpose of this surveillance system is to detect early warning signals of impending outbreaks and to help initiate an effective response in a timely manner. IDSP is a passive surveillance system that collects limited epidemiological data, does not collect data on immunization status of cases, and has limited involvement of the private sector and informal sectors [7].

The Indian Council of Medical Research—National Institute of Epidemiology (ICMR-NIE) has conducted sentinel surveillance for congenital rubella syndrome (CRS) since 2016. As of January 2024, there are 13 sentinel sites for CRS surveillance in the country.

Public health capacity is the ability to achieve stated public health objectives at the national, regional, and global levels with respect to both ongoing and emerging health problems [8]. Building the public health capacity is the process of improving the ability of the public health workforce to meet its objectives and to perform better. The public health workforce is the central component of the national public health capacity, which also includes infrastructural components such as resources, facilities, and appropriate technology [9].

Public health security is defined as the activities required, both proactive and reactive, to minimize the danger and impact of acute public health events that endanger people’s health [10]. The International Health Regulation (IHR) 2005 calls for a strengthened national capacity for surveillance and control; prevention, alert, and response to international public health emergencies beyond the traditional short list of required reporting; global partnership and collaboration; and rights, obligations, accountability, and procedures of monitoring [11].

India’s VPD surveillance program has enhanced both the public health capacity as well as the public health security. The aim of this article is to share insights into how the work towards strengthening and implementing comprehensive VPD surveillance has provided guidance for strengthening the national health capacity and national health security.

## 2. Methods

This article explains the processes related to the laboratory-supported VPD surveillance system in India conducted by the immunization division with the technical support of the WHO-NPSN.

### 2.1. VPD Surveillance Structure

A geographically well-distributed reporting network in the form of reporting units (RU) and informer units (IU) exists in the country.

1. Reporting units send regular weekly reports, including nil reports. As of January 2024, there are 12,022 reporting units in the country.
2. Informer units (IU) inform cases but do not send any weekly reports [3,4,5]. As of January 2024, there are 39,217 informer units in the country.

Taken together, more than 51,000 reporting sites have been established across the country. The reporting network involves both the public (~40%) and private (~60%) sectors, including Indian System of Medicine, i.e., AYUSH (Ayurveda, Yoga, Unani, Siddha, and Homeopathy), and other practitioners, based on the health-seeking behaviour of the community. Based on the potential of seeing suspected VPD cases, the reporting sites are classified into three priority categories—very high, high, and low. The VPD surveillance network stood the test of time during the COVID-19 pandemic as the reporting of AFP and FR cases continued during the pandemic.

### 2.2. Capacity Building of the Reporting Network

The reporting network is regularly sensitized for the identification and timely notification of suspected VPD cases. In 2023, approximately 5500 district and sub-district level workshops and >125,000 active case searches (ACSs) were conducted at these reporting sites. These ACS visits are important to assess the knowledge gaps existing among the health staff involved with VPD surveillance as well as to provide an opportunity to sensitize them. Surveillance posters and audio-visuals have been used to sensitize the health staff on the importance of VPD surveillance and overall surveillance processes.

### 2.3. Response to Reported VPD Cases

More than 90% of the suspected VPD cases get investigated within 48 h of notification by trained medical officers. The epidemiological data are collected through a case investigation form (CIF). Samples are collected as per the surveillance protocol and are shipped to the designated WHO-accredited laboratories in a timely manner under the required cold chain conditions.

### 2.4. Laboratory Network for VPD Surveillance

There are 58 VPD laboratories in the country for polio, MR, and diphtheria–pertussis (DP). These laboratories ensure the timely testing of samples to guide the surveillance and immunization program of the country. The WHO-NPSN with support from the government provides technical guidance and capacity building support for sample testing and quality assurance in these laboratories.

#### 2.4.1. Polio Laboratory Network

**Figure 2:**
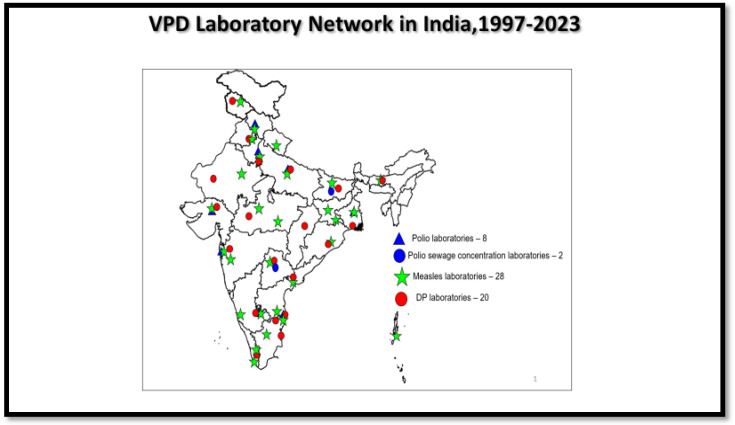
VPD laboratory network (polio, MR, and DP).

To maintain the achievement of the polio-free status, the country continues to have eight WHO-accredited polio laboratories since 1997 and two sewage sample concentration laboratories since 2016 (Figure 2). These polio laboratories are part of Global Polio Laboratory Network (GPLN).

#### 2.4.2. MR Laboratory Network

The MR laboratory network was established in 2005 based on the framework of polio laboratories and expanded to 28 laboratories to support the elimination program (Figure 2). Two of them are regional reference laboratories, seven are national laboratories, and nineteen are sub-national laboratories. The National Institute of Virology (NIV), Mumbai (for sequencing), and King Institute of Preventive Medicine (KIPM), Chennai (for serology), are designated as regional reference laboratories [12].

#### 2.4.3. DP Laboratory Network

The DP laboratory network was established in 2015 to support the surveillance program for the control of diseases and timely public health interventions. The gradual expansion of DP laboratories has been carried out with the expansion of DPT surveillance all over the country. As of 2023, there are 20 DP laboratories in the country, and the Christian Medical College (CMC) at Vellore has been designated to serve as the reference laboratory (Figure 2). These laboratories perform bacterial culture and molecular and serologic testing.

## 3. Results

Continuous efforts have been made to strengthen the VPD surveillance system in India, including the following:

- Identification and sensitization of potential informers (faith healers, temple sites, Indian System of Medicine [ISM] practitioners, and others): There has been an expansion of the reporting network to increase VPD surveillance reporting. From 41,800 reporting sites in 2015, the network has expanded to more than 51,000 reporting sites in the country.
- ∙ Capacity building of health functionaries: Between January 2020 and December 2023, more than 22,000 workshops on surveillance have been conducted, with more than 400,000 health personnel trained and oriented on VPDs through these cascaded training sessions across India. In 2023, approximately 5500 workshops were conducted at health facilities.
- Active case searches (ACSs) at the reporting sites: Between January 2020 and December 2023, more than 500,000 ACS visits have been made by government officers (District Immunization Officers/District Surveillance Officers/Surveillance Nodal Medical Officers) and WHO-NPSN Surveillance Medical Officers. In 2023 alone, more than 125,000 of these ACS visits were conducted at health facilities.
- Expansion of VPD laboratory network across the country: The expansion of surveillance has resulted in the expansion of both virology and bacteriology laboratories in the country. There is a strong component of quality assurance and quality control in these laboratories.
- Program review: Annually, two to three state-level VPD surveillance reviews are conducted in the country. These reviews have integrated the UIP reviews as well. Further, there is a close tracking of the progress of the immunization and surveillance program in the country through task force meetings, which are conducted periodically, usually monthly.
- Efficient data analysis and feedback mechanism: At the time of the initiation of AFP surveillance in 1997, data collation and analysis were mainly conducted using an Excel-based system. From 2005 to June 2023, the data management had been conducted through an offline epi-info based system called the Surveillance Information Management system (SIMS). From July 2023, the VPD surveillance data are being managed through a real time web-based tool called the ‘vaccine-preventable disease surveillance information management system (VSIMS)’.

As a result of the continuous efforts listed above, the VPD surveillance system has improved, as evident in the improvement of cardinal indicators of VPD surveillance mentioned below.

### 3.1. AFP Surveillance

The country continues to maintain and sustain the quality of AFP surveillance with indicators above the global recommended levels. The non-polio AFP rate for the country is well above the global recommended target (≥1 per 100,000 populations of less than 15 years of age). In 2023, the non-polio AFP rate was 5.8. Similarly, stool adequacy at the national level in 2023 was 89%—again above the global recommended target (≥80% of AFP cases, where two adequate stool specimens were collected within 14 days of paralysis onset) [13].

### 3.2. Measles and Rubella Surveillance

**Figure 3:**
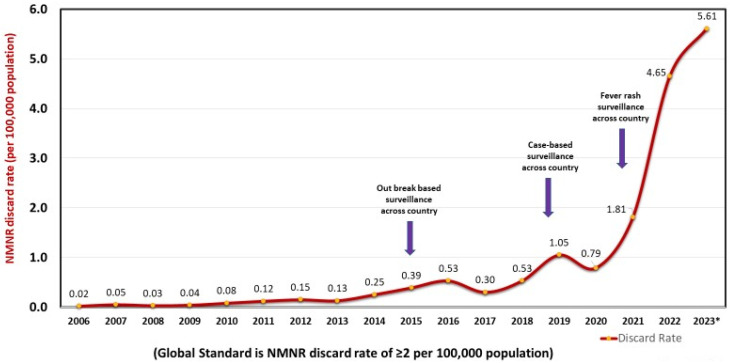
Non-measles–non-rubella discard rate (per 100,000 population), India, 2006–2023 *. * data as on 10 January 2024.

Over the years, MR surveillance has been strengthened in the country, especially as evidenced by the recent trend in non-measles–non-rubella (NMNR) discard rate, which is the globally accepted cardinal indicator for measuring MR surveillance sensitivity. The NMNR discard rate has shown tremendous progress, as a result of various surveillance strengthening efforts, and has improved from 0.53 in 2018 to 5.61 in 2023 (as of 10 January 2024) (Figure 3). This is two and half times greater than the global recommended standards of ≥2 per 100,000 population [14].

Additionally, the percentage of cases with adequate serum specimen collected within 28 days of rash onset was found to be above the global recommended target in 2023 (95% in 2023).

Measles–rubella outbreaks: The country’s sensitive surveillance has detected measles/rubella outbreaks in areas of immunity gaps. In 2023, of the 2225 suspected outbreaks investigated, 1215 were lab-confirmed measles outbreaks, and 13 were lab-confirmed rubella outbreaks [14]. Appropriate public health response measures were initiated, and more than 30 million children were vaccinated through MR campaigns and special immunization drives, including the outbreak response immunization in 2023.

With the enhancement of surveillance sensitivity, the number of fever-rash cases reported to the surveillance program increased from 33,379 in 2021 to 154,372 in 2023. Few of the case characteristics have been detailed in Table 2.

### 3.3. Diphtheria, Pertussis, and Neonatal Tetanus Surveillance

DPT surveillance guides the immunization program of the country.

Diphtheria: A total of 3724 suspected diphtheria cases have been reported in the country in 2023, compared to 3318 cases in 2022. Approximately 75% cases in 2022 and 80% cases in 2023 were in persons above five years of age. The overall incidence of suspected diphtheria cases at the national level was 2.3 and 2.7 per million population in 2022 and 2023, respectively.

Pertussis: A total of 4876 suspected pertussis cases have been reported in the country in 2023, compared to 4412 cases in 2022. Approximately 54% cases in 2022 and 63% cases in 2023 were in children below five years of age. The overall incidence of suspected pertussis cases at the national level was 2.3 and 2.7 per million population in 2022 and 2023, respectively.

Neonatal tetanus: Sixty-two cases of neonatal tetanus have been reported in the country in 2023, compared to the sixty-five cases reported in 2022. None of the districts in the country had neonatal tetanus cases above the elimination level, i.e., ≥1 NT case per 1000 live births during 2022 and 2023. Based on the surveillance data, the Standing Working Group on VPD surveillance under the National Technical Advisory Group on Immunization (NTAGI) has recommended that ‘a post-validation MNTE assessment should be considered in select districts which have reported several neonatal tetanus cases, even though the rate of disease is below the rate used to define MNTE’ [15].

### 3.4. VPD Laboratory Network

The laboratory network has immensely supported the VPD surveillance in the country. The timely provision of results by the laboratories has led to a timely response in the community. The number of samples processed by these laboratories over the years is shown below in Table 3.

## 4. Discussion

Continuous efforts have been made to strengthen the VPD surveillance system in India, which has resulted in enhancing the national health capacity and security. The best practices adopted are listed below.

### 4.1. Best Practices towards Health Capacity Enhancement

#### 4.1.1. Capacity Building of Health Personnel

High-quality cascaded training workshops with simulation exercises on the VPD outbreak investigation and response has helped in strengthening the outbreak response. The sensitization of frontline health workers, potential informers, and community leaders and the engagement of private health sector have strengthened the surveillance system, thereby improving the outbreak detection and response capacity. In 2023, 85% of the suspected diphtheria cases were notified within 7 days of onset of sore throat, and 95% of the notified cases were investigated within 48 h of notification. Approximately 95% of the notified suspected FR cases were investigated within 48 h, and adequate serological samples were collected in 97% of cases. In 2023, 1215 measles outbreaks were identified, and in 98% of these outbreaks, public health response activities were conducted, including outbreak response immunization.

#### 4.1.2. Ensuring Quality of Surveillance System at Par with the Global Standards

The verification and desk review of the case investigation forms of suspected VPDs by Surveillance Medical Officers of the WHO-NPSN, District Immunization Officers/Nodal Medical officers has ensured the reliable and quality data of VPDs to assess the disease burden. Such independent reviews of the investigation forms provide an extra layer of quality control for effective surveillance. These data are being used to provide guidance for policy decisions and to formulate evidence-based recommendations.

#### 4.1.3. Surveillance-Guided Program Planning and Implementation

Reliable and timely information is being provided through an effective and sensitive VPD surveillance for immediate public health response- and evidence-based policy decisions for program planning and implementation.

The surveillance data of AFP system helped in the identification of areas at high risk of polio transmission, which further helped in targeted mop-up campaigns, served as the final blow to interrupt the wild poliovirus (WPV) transmission. This has guided India to achieve and sustain their polio-free status since 13 January 2011. The best example is the development of the Kosi River operational plan to overcome the challenges in the access-compromised area (ACA) of Kosi River basin, which was the hub of WPV transmission in the state of Bihar. The plan was implemented successfully for several mop-up campaigns for polio, which resulted in a sharp decline in missed children during polio supplementary immunization activity (SIA) by the end of 2011 (1.4% missed children) as compared to 2008 (13.0% missed children) before the implementation of the plan. As a result, Bihar significantly reduced the transmission of WPV type 1 in the high transmission season and interrupted polio transmission in the main polio reservoir. With the help of this plan, the capacity was built to respond to exceptional circumstances like floods in the Kosi riverine area [16].

The AFP surveillance system has been leveraged to also support case-based, laboratory-supported measles–rubella surveillance. Based on the surveillance data, outbreak-based MR surveillance transitioned from the aggregate reporting of disease to case-based disease surveillance. MR surveillance has provided data for action in the form of policy decisions to introduce rubella-containing vaccines in the immunization program and to plan wide age-range MR SIAs across the country. As one of the key strategy, MR surveillance is guiding the progress towards the achievement of MR elimination in India [17].

The learned findings of the AFP and MR surveillance systems have been utilized to expand surveillance of other VPDs—diphtheria, pertussis and neonatal tetanus. The laboratory-supported surveillance data of diphtheria in 2017 and 2018 indicated a higher diphtheria burden in the age group of 5 years and above. The National Technical Advisory Group on Immunization (NTAGl) reviewed the surveillance data and recommended the introduction of the Td vaccine in UIP, replacing the TT vaccine in line with global recommendations, which was substantiated by the evidence of the age shift that was observed in diphtheria surveillance data. This resulted in the change from the TT vaccine to the Td vaccine in the adolescent age group of 10 years and 16 years as well as to pregnant women, a critical milestone in India’s effort to enhance the overall capacity of primary care and public health services [18].

#### 4.1.4. Strengthening of VPD Surveillance and Routine Immunization (RI) Program [19]

- High-risk areas identified through VPD surveillance were linked to RI session sites to improve immunization coverage;
- Advocacy and accountability framework through state, district/city, and block task forces for the polio program were utilized for other VPD surveillance and RI program reviews;
- VPD surveillance field reviews integrated the UIP field review to identify the gaps in the surveillance and routine immunization program, thereby strengthening VPD surveillance and RI;
- The identification and enlistment of areas vulnerable to MR outbreaks and carrying out the risk reprioritization of outbreak areas into high-risk areas for risk mitigation measures are crucial to prevent the outbreaks. This has resulted in the strengthening of the immunization and health system [20].

#### 4.1.5. Epidemic Response Team (ERT) at State/District/Block Levels

ERTs have been constituted at the state and district levels as a preparedness measure to rapidly respond to VPD outbreaks. At the state level, the State Immunization Officer, State Surveillance Officer from IDSP, and Sub-regional Team Leader and, at the district level, the District Immunization Officer (DIO), District Surveillance Officer (DSO), and Surveillance Medical Officer of NPSN are providing direct oversight to the state and district ERTs, respectively. The constitution and effective coordination of all the key players of the ERT at all levels for immediate public health responses have been found to be critical towards initiating early investigations and implementing public health response measures. During the COVID-19 pandemic, the WHO-NPSN SMOs coordinated at the district and block levels to mobilize the ERT to support contact tracing, home quarantine, and home isolation.

#### 4.1.6. VPD Surveillance System Backed by an Effective Network of Diagnostic Laboratories

Laboratory networks play a pivotal role in the accurate and timely confirmation of infections supporting public health response to the disease and thus become an essential component of the disease surveillance system. These laboratory networks guide programs for disease control, elimination, and eradication [21].

An efficient AFP and environmental surveillance system for poliovirus detection has continuously been supported by the WHO-accredited laboratory network for the virological diagnosis of polioviruses. Considering the sensitivity of environmental surveillance in detecting the silent transmission of WPV/vaccine-derived poliovirus (VDPV), ES has been expanded further in Rajasthan, Punjab, Jammu, and Kashmir in 2022–2023, all near the international border with Pakistan, which is still endemic for WPV1. ES was successfully used to demonstrate the end of WPV transmission in the country as well as to judge the effectiveness of polio immunization campaigns, and post polio-free certification, it is used as supplementary surveillance to screen presence of any WPV/VDPV in the country [22,23,24,25].

Twenty-eight MR laboratory networks are supporting laboratory-based surveillance to reach MR elimination goal in the country, while 20 bacteriology laboratories are supporting the control of diphtheria–pertussis. The diagnosis and case classification through the results obtained from these laboratory support programs take quick actions for mass immunization or develop additional strategies to control the infection/outbreaks.

Nearly all the VPD laboratories belong to the public sector and have the capacity to detect the viral and bacteriological pathogens, as necessitated.

#### 4.1.7. Leveraging VPD Surveillance System for COVID-19 Response and Other Public Health Priorities

The efforts towards nurturing VPD surveillance in the country that included the expansion of the reporting network, capacity building, case notification, sample collection, and shipment proved immensely useful during the COVID-19 pandemic. The existing VPD surveillance and IDSP platforms were utilized effectively to operationalize the collection of case data during the recent COVID-19 pandemic. The medical officers involved with the investigation of VPD cases were trained to investigate the COVID-19 cases and collect information on a case investigation form that was developed for influenza-like illness (ILI) and severe acute respiratory infection (SARI) [26].

In 11 cities of the country, the environmental surveillance sites for poliovirus detection were leveraged for the detection of SARS CoV-2 RNA. Eight polio labs were involved with testing sewage samples for SARS CoV-2, in addition to testing for polioviruses. The evidence generated through this surveillance demonstrated a linear relation between the increase in the load of COVID-19 cases detected through case-based surveillance and positivity in the sewage sample, thereby demonstrating the utility of the system.

As a part of the COVID-19 surveillance strengthening efforts, laboratories played a pivotal role in surveillance while maintaining good quality within the laboratory network, which helped in identifying control measures for the disease. The WHO supported the quality of laboratory network operating under the Ministry of Health and Family Welfare, the Indian Council of Medical Research, and the National Centre for Disease Control by providing an external quality assurance panel (EQAP) [27]. Out of 1312 laboratories which participated, 91% qualified by achieving ≥80% scores, thus assuring the quality of testing under these laboratories for COVID-19 detection.

These measures led to a timely detection of COVID-19 cases and guided the implementation of rapid public health response measures.

The task forces established earlier for polio elimination at the district and state levels were utilized for the COVID-19 pandemic and ensured good intersectoral coordination for effective public health response measures, including for COVID-19 vaccination coverage.

Taking the learned findings of VPD surveillance and routine immunization, training modules were developed for capacity building of the stakeholders for the COVID-19 vaccination program at all levels. Cascaded training was conducted across the country to build the capacity of health staff. India has extended support to other countries through capacity building and the sharing of their experience on the rollout of the COVID-19 vaccine.

Other public health priorities: During 2016–2022, under the EIS and FETP, the WHO-NPSN network provided support in detailed epidemiologic investigations of the select outbreaks of cholera, acute diarrheal disease, dengue, chicken pox, typhoid, malaria, hepatitis A, H1N1, and Kyasanur Forest Disease (KFD). VPD surveillance tools were used to strengthen KFD surveillance in the vulnerable districts of Karnataka. The capacity of the local government was built for surveillance and outbreak responses, including conducting vaccination campaigns for KFD control [28].

The NPSN personnel have also been involved in flood/cyclone responses in the states of Kerala, Odisha, and Tamil Nadu in recent years. The staff supported the public health response to communicable diseases during the floods and also strengthened surveillance for communicable diseases in these areas.

### 4.2. Best Practices towards Health Security Enhancement

#### 4.2.1. Governance Mechanism and Intersectoral Coordination

With strong governance in the form of task forces at various levels and good intersectoral coordination, the strategies of polio eradication were effectively implemented that led to the polio-free status in India. The findings were carried forward to track and review the MR elimination progress and to strengthen outbreak preparedness and response measures for other VPDs like MR, diphtheria, and pertussis.

#### 4.2.2. Cross-Border Cooperation

Infectious diseases do not respect international borders. Cross-border coordination for vaccination strategies and surveillance proved essential for the eradication of polio. The best example is the expansion of environmental surveillance sites for poliovirus detection in select cities along the Indo-Pakistan border and the vaccination sites along the borders of the country with neighboring countries, notably Bangladesh, Nepal, and Pakistan.

#### 4.2.3. Data Management System

An efficient and robust data management system is in place with the capacity to detect impending outbreaks through early warning signals for immediate public health responses. The triangulation of vaccination coverage and VPD surveillance data helps to better understand the immunity gaps in the population and support policy decisions targeted towards strengthening the immunization system.

#### 4.2.4. Support in Public Health Emergencies of National and International Concern

The capacity built over the years among the VPD surveillance staff in India has been used during public health emergencies outside the country such as during the Ebola outbreak in Western Africa, polio outbreak in Nigeria, and diphtheria outbreak in Cox’s Bazar. The same has been replicated in India, most recently with regard to the COVID-19 pandemic. An adequate VPD surveillance system backed by an effective network of WHO-accredited diagnostic laboratories enhance the country’s capacity to detect and manage other communicable diseases of public health concern.

## 5. Strengths and Limitations of the Surveillance System

Strengths: The surveillance system for VPDs in India extends across the length and breadth of the country, covering every district of every state and union territory of the country. The reporting network for VPDs includes health facilities both from the public and private sectors and can be adjusted based on the health-seeking behavior of the community to individual diseases. The system is a case-based, laboratory-supported system that is able to generate detailed information on cases and outbreaks of individual diseases that contributes to the control/elimination of such diseases. The surveillance system has a built-in component of active surveillance that improves the reporting of the VPDs that are under the surveillance. The data management and analysis component of the system not only ensures collation of the large data base but also ensures the availability of real-time information that can be put to programmatic action at the state and district levels, in addition to policy decision making at the national level. Indicators to measure the sensitivity of the system are well defined and used to assess the same at the national and subnational levels, based on which gaps in quality can be identified and appropriate changes made to enhance quality. The system has ensured building national capacity for surveillance and public health response to several diseases, even those outside of the scope of the current system.

Limitations: The surveillance system is resource intensive and needs dedicated staff and finances to support the field implementation and laboratory testing of samples. Integration with IDSP will require a revision in the protocols of IDSP so that the system can serve the needs for VPDs, especially those that are targeted for elimination.

## 6. Conclusions

India initiated VPD surveillance with AFP surveillance in 1997, and over the years, AFP surveillance has been strengthened and environmental, MR surveillance, other VPDs like diphtheria, pertussis, and neonatal tetanus have been integrated into this surveillance system. The geographically well-distributed WHO-accredited/proficient VPD surveillance laboratories are an integral component of the surveillance system.

India’s experience with VPD surveillance strengthening efforts has guided the immunization program. It has enhanced the national health capacity and security in terms of detection and response to VPDs, thereby contributing to the eradication of polio, the elimination of maternal and neonatal tetanus, efforts towards the elimination of measles and rubella, and the control of diphtheria, pertussis, and other VPDs. The efforts made to maintain the VPD surveillance system have the potential to contribute to support the surveillance of other diseases, thereby enhancing national health capacity and security.

## Figures and Tables

**Table 1:** National immunization schedule—India [3].

Age	Vaccines Given
Birth	BCG, OPV 0, Hepatitis B birth dose
6 weeks	OPV 1, Pentavalent 1 *, Rotavirus 1, fractional IPV 1, PCV 1
10 weeks	OPV 2, Pentavalent 2, Rotavirus 2
14 weeks	OPV 3, Pentavalent 3, Rotavirus 3, fractional IPV 2, PCV 2
9–12 months	MR 1, JE 1 **, PCV booster, fIPV 3
16–24 months	MR 2, JE 2 **, DPT booster 1, OPV booster
5–6 years	DPT booster 2
10 years	Td
16 years	Td
Pregnant mother	Td 1, Td 2, Td booster ***

OPV: oral poliovirus vaccine; IPV: inactivated poliovirus vaccine; PCV: pneumococcal conjugate vaccine; MR: measles–rubella vaccine; JE: Japanese Encephalitis vaccine; DPT: diphtheria, pertussis, and tetanus vaccine; Td: tetanus and adult diphtheria. * Pentavalent: diphtheria, pertussis, tetanus, hepatitis B, and *Hemophilus influenzae* type b. ** JE in endemic districts only. *** One dose if previously vaccinated within three years.

**Table 2:** Distribution of fever–rash cases by age, sex, and setting, 2021–2023.

		2021	2022	2023
Fever–rash cases		33,379	112,226	154,372
Age group	<5 years	50%	53%	48%
	5–10 years	20%	24%	26%
	>10 years	30%	23%	26%
Sex	Male	56%	55%	55%
	Female	44%	45%	45%
Setting	Rural	71%	64%	69%
	Urban	29%	36%	31%

**Table 3:** Sample testing load at VPD surveillance laboratories.

Year	1997–2023	2005–2023	2006–2023	2014–2023	2019–2023
Polio (AFP)	1,721,408				
Polio (ES)		17,100			
MR (Serology)			309,200		
MR (Molecular)				161,364	
Diphtheria (TS)					16,985
Pertussis (NPS and Serology)					23,256

AFP, acute flaccid paralysis; ES, environmental surveillance; TS, throat swab; NPS, nasopharyngeal swab.

## Data Availability

The raw data supporting this article will be made available by the authors without undue reservation.
